# The prognostic value of separate lymphatic invasion and vascular invasion in oesophageal squamous cell carcinoma: a meta-analysis and systematic review

**DOI:** 10.1186/s12885-022-10441-6

**Published:** 2022-12-19

**Authors:** An Wang, Yulong Tan, Shaohua Wang, Xiaofeng Chen

**Affiliations:** Department of Thoracic Surgery, Huashan Hospital, Fudan University, Shanghai, China

**Keywords:** Oesophageal carcinoma, Lymphatic invasion, Vascular invasion, Prognosis

## Abstract

**Background:**

Lymphovascular invasion (LVI) is a factor correlated with a poor prognosis in oesophageal squamous cell carcinoma (ESCC). Lymphatic invasion (LI) and vascular invasion (VI) should be reported separately because they may indicate a difference in prognosis. The prognostic role of LI and VI in ESCC patients remains controversial. A meta-analysis was conducted to resolve this question.

**Methods:**

We searched the PubMed, EMBASE, Web of Science, Scopus and Cochrane Library databases for studies on the association between LI and VI and the prognosis of patients with ESCC. The PICOs (Participant, Intervention, Comparison, Outcome) strategy were selected for the systematic review and meta-analysis. The effect size (ES) was the hazard ratio (HR) or relative ratio (RR) with 95% confidence intervals (CI) for overall survival (OS) and recurrence-free survival (RFS).

**Results:**

A total of 27 studies with 5740 patients were included. We calculated the pooled results from univariate and multivariate analysis using the Cox proportional hazards method. The heterogeneity was acceptable in OS and RFS. According to the pooled results of multivariate analysis, both LI and VI were correlated with a worse OS. VI was a negative indicator for RFS, while the *p value* of VI was greater than 0.05. The prognostic role was weakened in subgroup analysis with studies using haematoxylin–eosin staining method.

**Conclusions:**

Both LI and VI were indicators of a worse OS outcome. LI was a more significant indicator in predicting a worse RFS. More larger sample studies with immunohistochemical staining and good designs are required to detect the prognostic value of separate LI and VI in ESCC.

**Supplementary Information:**

The online version contains supplementary material available at 10.1186/s12885-022-10441-6.

## Introduction

Oesophageal cancer (EC) morbidity is increasing worldwide and it is the seventh most common malignant cancer in the world [1]. The mortality from EC is the sixth leading cause of cancer-related death worldwide, with a low 5-year survival rate of EC patients ranging from 15%-34% [1, 2]. Oesophageal squamous cell carcinoma (ESCC) is one of the main types of EC. The most important prognostic factors are tumour characteristics (such as tumour size, tumour location, depth of invasion, differentiation) and whether there are any affected regional lymph nodes and/or metastatic sites (nonregional lymph nodes and organs outside the oesophagus) according to the 8th AJCC Cancer Staging Manual [3]. Our previous studies suggested that lymphovascular invasion, which was correlated with the ability of the cancer to metastasize, was associated with a poor prognosis in EC patients [4–6].

Lymphovascular invasion (LVI) refers to the presence of malignant cells within lymph vessels and/or vascular vessels. The distinction between lymphatic invasion and vascular invasion could be made by the presence of erythrocytes in the endothelial line and a thick vessel wall [7]. Lymphatic invasion and vascular invasion should be reported separately because they may indicate a difference in prognosis on the basis of the 8th AJCC Cancer Staging Manual [3]. However, studies about the prognostic role of LI and VI were controversial. Sarbia et al. reported that LI and VI were both poor prognostic indicator [8]. Waraich et al. reported that VI is not a prognostic indicator of recurrence [9]. Additionally, the prognosis of thoracic ESCC patients with both LI and VI was worse than that of patients with LI or VI alone [10]. However, there is no study with a large sample concentrating on the prognostic value of lymphatic invasion and vascular invasion in ESCC separately. Therefore, we conducted this meta-analysis and systematic review to evaluate the relationship between separate lymphatic invasion, vascular invasion and prognosis in ESCC patients.

## Materials and methods

This meta-analysis and systematic review were carried out in line with the Preferred Reporting Items for Systematic Review and Meta-Analysis guidelines [11].

### Search strategy

PubMed, EMBASE, Web of Science, Scopus and Cochrane Library databases were searched for relevant studies published through Nov. 4, 2022. The combination of search bar was: ((((lymphovascular invasion OR lymph vessel invasion OR angiolymphatic invasion OR lymphatic invasion OR lymphangiogenesis OR venous invasion OR vascular invasion OR blood vessel invasion)) AND (esophageal cancer OR esophageal carcinoma)) AND (survival OR prognosis)). Only studies published in English were reviewed.

### Selection criteria and data extraction

The PICOs (Participant, Intervention, Comparison, Outcome) strategy were adopted for the selection of studies. The preliminary screen was carried out by title and abstract on the basis of PICOs. Exclusion criteria included: (1) duplicate reports, letters, conference papers, and reviews, (2) studies that did not contain prognostic information, (3) oesophagogastric junction cancer (EJC), and (4) sample size less than 100 patients. Inclusion criteria: (1) the histology type of cancer was ESCC, (2) included articles published in English, (3) high quality of included studies and (4) studies included in this meta-analysis must provide sufficient survival data about LI and VI, and (5) patients must undergo operation. The prognostic indicator was hazard ratio (HR) or relative ratio (RR). The prognostic outcome was overall survival (OS) or relapse-free survival (RFS). The prognostic outcome was analysed with the Cox proportional hazards method. If studies were published on the same group of patients, the newest or the most informative article was selected. The K agreement between the reviewers is 0.76. The K agreement was based on measurement consistency assessment in the Cochrane handbook for systematic reviews of interventions [12].

### Quality assessment of included studies

To ensure the quality of the included studies, the criteria derived from Reporting Recommendations for Tumor Marker Prognostic Studies (REMARK) were used to assess the included studies [13]. The selected criteria derived from REMARK guidelines were listed in Table 1.

**Table 1:** Criteria used to elevate the quality of included studies in this meta-analysis of six most reported biomarkers (derived from REMARK)

Checklist	Criteria
1-Samples	Cohort study with a well-defined study population. Medical treatment for patients was reasonable. Authors provided justification for excluded patients
2- Clinical parameters of the cohort	The clinical parameters such as age, gender, surgical procedure, pathologic stage and histological type was provided
3-Staining Method	Clear staining method such as H&E and IHC was referred to original paper
4-Prognosis	The survival endpoints were defined as overall survival and disease-free survival
5- Statistics	Statistical analysis was univariate or multivariate Cox proportional hazards method
6- Effect size	HR or RR with 95% CI and *P*-vales were provided

*Abbreviations*: *H&E* Haematoxylin–eosin, *IHC* Immunohistochemistry, *CI* Confidence interval, *RR* Relative risk, *HR* Hazard risk

### Risk of bias appraisal

The risk of bias of included studies was assessed by the Cochrane Risk of Bias Assessment Tool [14]. This tool contains nine domains: representativeness of the exposed cohort; selection of the non-exposed cohort; ascertainment of exposure; outcome was not present at start of study; comparability of cohorts on the basis of the design or analysis (main and other confounding factors were not statistically different); assessment of outcome; enough follow-up; adequacy of follow up of cohorts. Each domain was scored as “high risk of bias”, “low risk of bias”, or “unclear risk of bias”. The plot of risk of bias was generated by Review Manger 5.3.

### Data extraction

Potential articles were independently reviewed by 2 investigators (Wang A. and Tan Y.) against the above criteria. Disagreements were discussed and resolved by a third author (Wang S.). Data from the included studies were independently extracted by two authors (Wang A. and Tan Y.) using a standardized form. A third investigator (Wang S.) checked the collected data for accuracy. The following information was extracted: surname of the first author, years included, country, sample size of included studies, patients’ characteristics, stage information, staining method, number of LI and VI, survival statistics, statistical method and compliance to REMARK criteria.

### Statistical analysis

Statistical analysis was performed using Stata/SE version 12.0 for Windows (Stata Corporation, College Station, TX, USA). A worse prognosis of ESCC was indicated by pooled HR value > 1. Cochran’s Q test (Chi-squared test; Chi^2^) and the I^2^ metric were performed to test the heterogeneity of the pooled results. An I^2^ value less than 25% indicated no heterogeneity; an I^2^ value between 25 and 50% suggested moderate heterogeneity; an I^2^ value between 50 and 75% suggested medium heterogeneity; and an I^2^ greater than 75% was considered extreme heterogeneity. We adopted a fixed-effects model (the Mantel–Haenszel method) when I^2^ < 50% with *p* > 0.05 in this meta-analysis. If not, we used a random-effects model. Subgroup analysis was used to explore heterogeneity when necessary. Begg’s test was used to assess publication bias. Two-tailed tests were adopted to calculate the *p* value, and *p* ≤ 0.05 was considered statistically significant.

## Results

### Characteristics of the studies

**Fig. 1:**
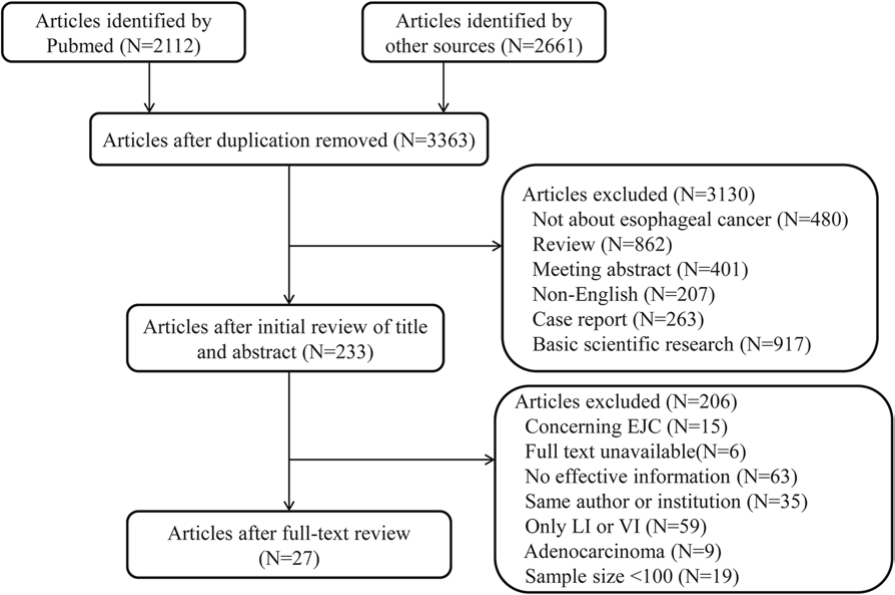
Flow chart showing the literature review procedure for the included studies

We retrieved 3363 articles after removing the duplicates. A total of 3130 articles were excluded after screening the titles and abstracts. We identified 233 potential articles for full-text review. Finally, 27 articles were eligible for this meta-analysis after 206 articles were excluded. The detailed information of the study inclusion process is listed in the flow chart (Fig. 1).

The remaining 27 articles, which included information on 5740 patients (range: 101–863), were included in the meta-analysis. All of the extracted information was listed in Table 2. A total of five studies adopted RR as the prognostic indicator [8, 15–18]. The prognostic indicator of the remaining studies was HR.

**Table 2:** Characteristics of studies included in our meta-analysis

**Author**	**Years Included**	**Country**	**No**	**Patients’ Characteristic**	**Stage Method**^a^**/Stage/ (I + II ≥ 60%)**	**Staining Method**	**Indicator (No.)**	**Survival Statistics**	**Statistical Method**	**Compliance to REMARK criteria**
Ma (2022) [18]	2013–2017	China	396	Oesophagectomy and lymphadenectomy (118 right incision, 278 left incision), no NAT, 210 N0, 186 N + , some patients received PAT	8^th^ TNM/I-IV/No	IHC	VI(102)/LI(94)	RFS	Mul	Fulfilled items
Toriumi (2022) [19]	2008–2017	Japan	154	Esophagectomy with lymphadenectomy, no NAT, 103 N0, 51 N + , no PAT information	8^th^ TNM/I-IV/Yes	H&E/IHC	VI(97)/LI(46)	OS/RFS	Mul and Uni	Checklist no.2 was not fulfilled
Kitamura (2021) [20]	2010–2015	Japan	327	McKeown oesophagectomy with lymph node dissection (189 2-field, 134 3-field), 212 patients received NAT, no PAT information, 159 N0, 168 N +	8^th^ TNM/I-IV/Yes	NM	VI(129)/LI(135)	OS	Mul and Uni	Checklist no.3 was not fulfilled
Baba (2020) [21]	2001–2016	Japan	147	Radical esophageal resection, 70 patients received NAT, some patients received PAT	8^th^ TNM/I-III/No	IHC	VI(NM)/LI(NM)	RFS	Mul and Uni	Checklist no.2 was not fulfilled
Oguma (2019) [22]	1999–2016	Japan	195	Thoracic oesophagecttomy with 3-field lymphadenectomy, no NAT, some patients received PAT	JCEC, 11^th^ edition/T1N0M0/Yes	H&E	VI(57)/LI(110)	RFS	Mul and Uni	Fulfilled items
Arigami (2018) [23]	1998–2012	Japan	237	Oesophagectomy with Lymph node dissection, no NAT, 113 N0, 124 N + , no PAT information	JCEC, 11^th^ edition /I-III/Yes	NM	VI(138)/LI(143)	OS	Mul and Uni	Checklist no.2 and no.3 was not fulfilled
Nakamura (2017) [24]	2005–2016	Japan	245	Oesophagectomy, no NAT, 182 N0, 63 N + , 199 PAT	7^th^ TNM/T1N0-3/Yes	NM	VI(75)/LI(71)	RFS/OS	Mul and Uni	Checklist no.2 and no.3 was not fulfilled
Okada (2016) [25]	1997–2011	Japan	160	Transthoracic or transhiatal approach performed in 143 and 17 patients, minimally invasive thoracoscopic surgery performed in 10 cases, all patients received 3-field lymphadenectomy, 58 N0, 102 N + , 67 NAT, no PAT information	6^th^ TNM II-III/Yes	H&E	VI(138)/LI(86)	RFS/OS	Mul and Uni	Fulfilled items
Han (2016) [26]	2007–2008	China	218	Ivor-Lewis and the three-stage (right thoracotomy, midline laparotomy and left cervical incisions) esophagectomy, no NAT, 128 N0, 90 N + , 78 PAT	7^th^ TNM/I-III/Yes	IHC	VI(35)/LI(58)	RFS/OS	Mul and Uni	Fulfilled items
Takata (2014) [27]	1998–2007	Japan	191	Oesophagectomy via a right thoracotomy, with a two or three field lymphadenectomy, 86 NAT, 68 N0, 123 N + , no PAT information	7^th^ TNM/I-IV/No	NM	VI(79)/LI(148)	OS	Mul and Uni	Checklist no.3 was not fulfilled
Takeno (2013) [28]	1991–2010	Japan	228	Radical oesophagectomy with 3-field lymph nodes dissection, no NAT, 112 N0, 116 N + , no PAT information	7^th^ TNM/I-IV/Yes	NM	VI(68)/LI(130)	OS	Mul	Checklist no.3 was not fulfilled
Nakashima (2013) [15]	1994–2005	Japan	101	Oesophagectomy, no NAT, 50 N0, 51 N + , no PAT information	JCEC, 9^th^ edition/T1-4N0-3/Unclear	H&E	VI(37)/LI(47)	OS	Mul and Uni	Checklist no.2 was not fulfilled
Ichikawa (2013) [29]	1995–2010	Japan	315	Radical thoracoscopic-assisted oesophagectomy and lymphadenectomy, 74 NAT, 134 N0, 181 N + , 44 PAT	6^th^ TNM/IIa-IV/No	NM	VI(210)/LI(199)	OS	Uni	Fulfilled items
Kim (2012) [30]	NM	Korea	138	Oesophagectomy with lymph node dissection, no NAT, 47 N0, 91 N + , no PAT information	7^th^ TNM/I-IV/No	H&E	VI(21)/LI(80)	OS	Mul	Fulfilled items
Suzuki (2011) [31]	1990–2007	Japan	138	Curative surgery, no NAT, 55 N0, 83 N + , no PAT information	6^th^ TNM/I-IV/No	NM	VI(88)/LI(109)	OS	Mul and Uni	Checklist no.2 and no.3 was not fulfilled
Ren (2010) [16]	2003–2004	China	148	Radical oesophagectomy, no NAT, 74 N0, 74 N1, no PAT information	6^th^ TNM/T1-4N0-1M0-1/Unclear	NM	VI(69)/LI(80)	OS	Uni	Checklist no.2 and no.3 was not fulfilled
Tateno (2009) [32]	1987–1998	Japan	216	Oesophagectomy with lymph node dissection, no NAT, 77 N0, 139 N1, no PAT information	5th TNM/I-IV/No	IHC	VI(77)/LI(136)	OS	Mul	Fulfilled items
Sano (2009) [33]	1995–2005	Japan	151	Curative surgical resection, no NAT, 66 N0, 85 N1, no PAT information	6^th^ TNM/I-IV/No	H&E	VI(89)/LI(113)	RFS	Mul and Uni	Checklist no.2 was not fulfilled
Komatsu (2009) [34]	1981–1995	Japan	153	Surgery, no NAT, 34 N0, 119 N1, no PAT information	6^th^ TNM/I-IV/No	H&E	VI(113)/LI(135)	OS	Uni	Checklist no.2 was not fulfilled
Tsujitani (2007) [35]	1981–1995	Japan	107	Oesophagectomy with lymph node dissection, no NAT, 44 N0, 63 N + , some patients received PAT	5^th^ TNM/0-IV/Unclear	H&E	VI(23)/LI(64)	OS	Mul	Fulfilled items
Natsugoe (2007) [36]	1988–1998	Japan	194	Oesophagectomy with lymph node dissection, no NAT, 84 N0, 110 N1, no PAT information	5^th^ TNM/I-IV/No	NM	VI(63)/LI(114)	OS	Mul	Checklist no.3 was not fulfilled
Dhar (2007) [37]	1998–2003	Japan	863	Right thoracotomy with two-field or three-field LN dissection, no NAT, 572 N0, 291 N + , 81 PAT	6^th^ TNM/I-IV/No	H&E	VI(NM)/LI(NM)	OS	Mul	Fulfilled items
Takahashi (2006) [38]	1990–2000	Japan	180	Resection of the oesophagus with lymph node dissection, no NAT, 90 N0, 90 N + , no PAT information	6^th^ TNM/0-IV/Unclear	NM	VI(39)/LI(83)	OS	Mul	Checklist no.2 and no.3 was not fulfilled
Kato (2002) [39]	1989–1999	Japan	130	Radical total oesophagectomy and three- field lymph node dissection, 9 R1, no NAT, 64 N0, 66 N1, 52 PAT	5^th^ TNM/I-IV/Yes	H&E	VI(43)/LI(71)	OS	Uni	Fulfilled items
Osugi (2002) [17]	1986–1998	Japan	247	Standard three-stage oesophagectomy and three- field lymph node dissection, no NAT, 88 N0, 159 N + , no PAT information	JCEC, 9^th^ edition/Tis-4N0-3M0/Unclear	H&E	VI(76)/LI(161)	OS	Mul	Fulfilled items
Tachibana (1999) [40]	1979–1998	Japan	129	Right transthoracic subtotal oesophagectomy with three-field lymph node dissection, no NAT, 62 N0, 67 N + , some patients received PAT	5^th^ TNM/0-IV/No	NM	VI(19)/LI(43)	OS	Mul	Fulfilled items
Sarbia (1995) [8]	1978–1992	Germany	161	Subtotal oesophageal resection, no NAT, no PAT information	4^th^ TNM/NM/NM	H&E	VI(53)/LI(78)	OS	Mul	Fulfilled items

*Abbreviations*: *NAT* Neoadjuvant treatment, *PAT* Postoperative adjuvant treatment, *LNM* Lymph node metastasis, *H&E* Haematoxylin–eosin, *IHC* Immunohistochemistry, *LI* Lymphatic invasion, *VI* Vascular invasion, *NM* Not mentioned, *OS* Overall survival, *RFS* Recurrence-free survival, *RT* Radiotherapy, *Mul* Multivariate, *Uni* Univariate, *JCEC* Japanese Classification of Oesophageal Cancer

^a^The staging method was based on AJCC/UICC, if no special instructions were mentioned

**Fig. 2:**
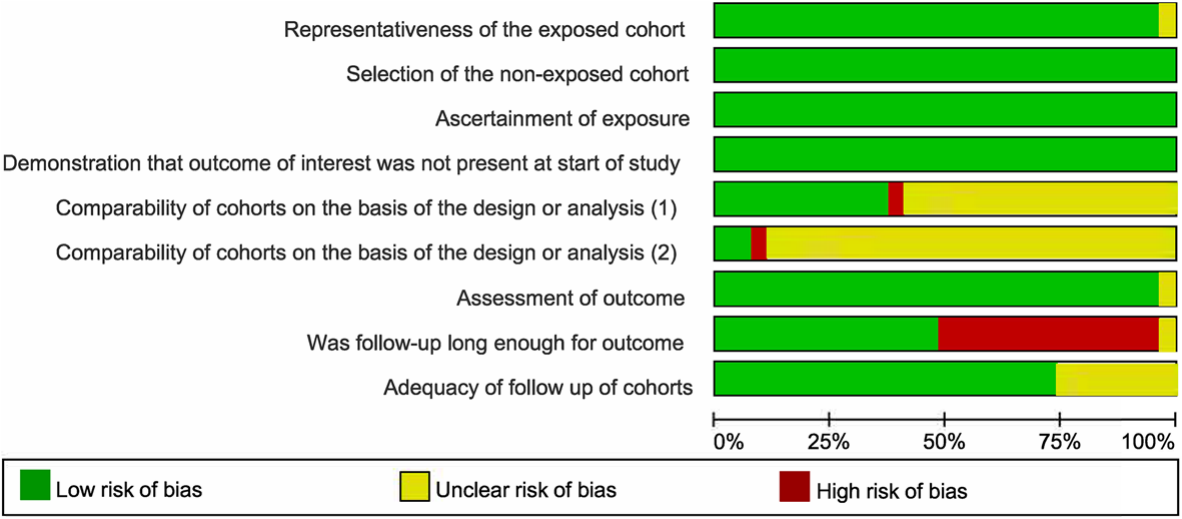
The risk of bias graph. (1): The age of the two groups is not statistically significant. (2): Other important confounding factors were not statistically different

The risk of bias was assessed by the Cochrane Risk of Bias Assessment Tool. And the results were displayed in Fig. 2. The quality of included studies was elevated by REMARK guidelines. Thirteen studies met all the checklists. Checklist no.2 or no.3 could not be fulfilled in the rest studies.

The prognostic outcomes were OS and RFS. Both the multivariate and univariate Cox proportional hazards methods were used by all of the included studies. All pooled survival outcomes were calculated by multivariate and univariate Cox proportional hazards methods. The results are listed in Tables 3 and 4.

**Table 3:** Pooled results of multivariate analysis

Indicator	LI		Heterogeneity analysis		Publication bias	VI		Heterogeneity analysis		Publication bias
	HR (95% CI)	*p* value	I^2^	*p* value		HR (95% CI)	*p* value	I^2^	*p* value	
OS	1.33 (1.17–1.50)	< 0.0001	4.1%	0.407	0.017	1.29 (1.16–1.45)	< 0.0001	24.5%	0.166	0.596
RFS	1.71 (1.24–2.36)	0.001	50.5%	0.049	0.063	1.28 (1.07–1.52)	0.07	0%	0.682	1

**Table 4:** Pooled results of univariate analysis

Indicator	LI		Heterogeneity analysis		Publication bias	VI		Heterogeneity analysis		Publication bias
	HR (95% CI)	*p* value	I^2^	*p* value		HR (95% CI)	*p* value	I^2^	*p* value	
OS	2.08 (1.81–2.38)	< 0.0001	46.5%	0.033	0.127	1.80 (1.60–2.02)	< 0.0001	18.4%	0.258	0.583
RFS	2.62 (1.81–3.78)	< 0.0001	56.4%	0.033	0.035	1.91 (1.53–2.38)	< 0.0001	0%	0.798	0.368

### Overall survival

**Fig. 3:**
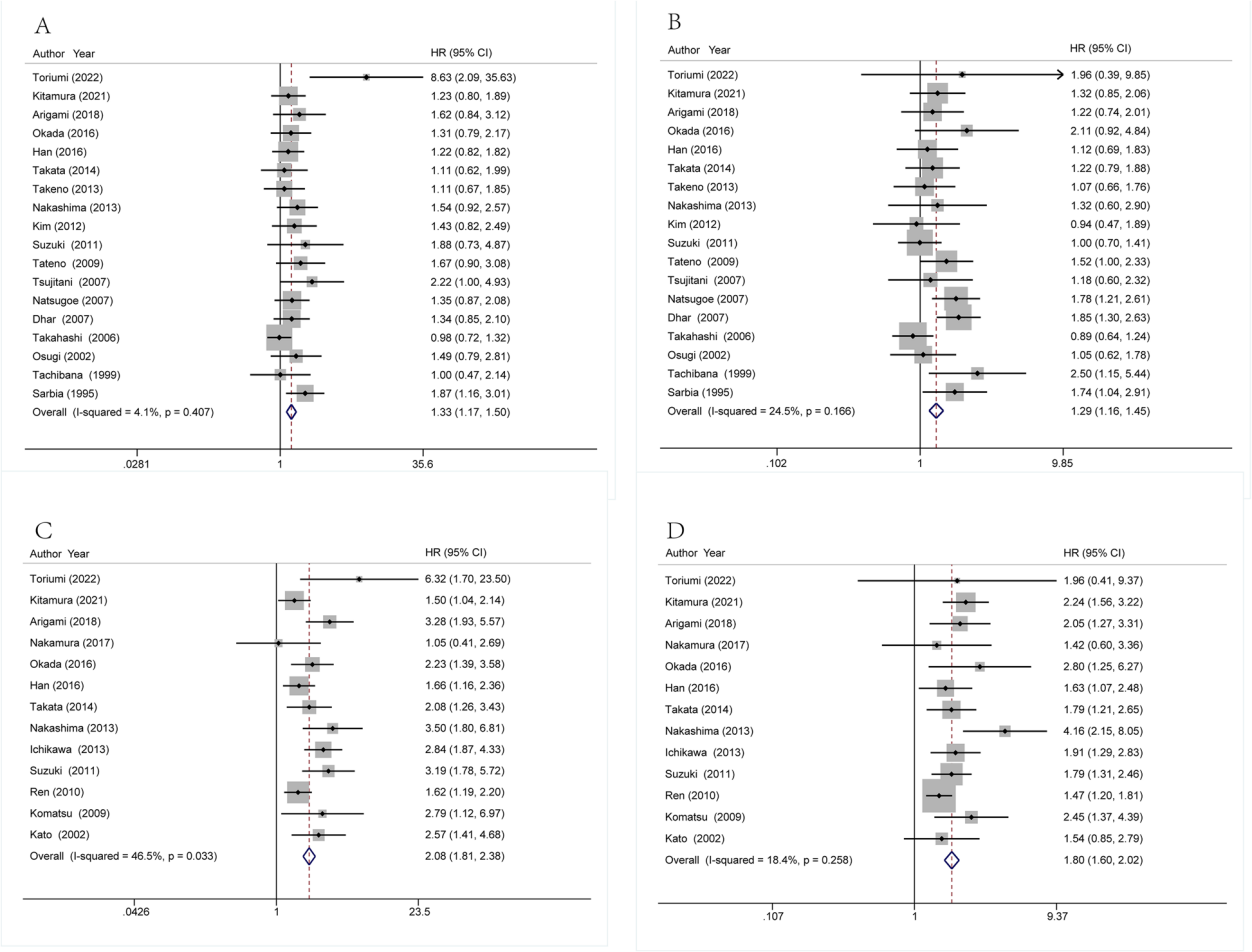
Forrest plot showing the pooled HR for OS, (**A**) LI from multivariate analysis, (**B**) VI from multivariate analysis, (**C**) LI from univariate analysis, (**D**) VI from univariate analysis

Eighteen studies containing 3989 ESCC patients provided multivariate HR. The pooled multivariate HRs with 95% CIs for LI and VI were 1.33 (1.17–1.50, *p* < 0.0001) and 1.29 (1.16–1.45, *p* < 0.0001), respectively. The I^2^ for LI and VI was 4.1% (Chi^2^ = 17.72, *p* = 0.407) and 24.5% (Chi^2^ = 22.5, *p* = 0.166), respectively. Thirteen studies concerning 2465 patients provided univariate HR. The pooled univariate HRs with 95% CIs for LI and VI were 2.08 (1.81–2.38, *p* < 0.0001) and 1.80 (1.60–2.02, *p* < 0.0001), respectively. The I^2^ for LI and VI was 46.5% (Chi^2^ = 22.43, *p* = 0.033) and 18.4% (Chi^2^ = 14.70, *p* = 0.258), respectively. The pooled results are shown in Fig. 3.

### Recurrence-free survival

**Fig. 4:**
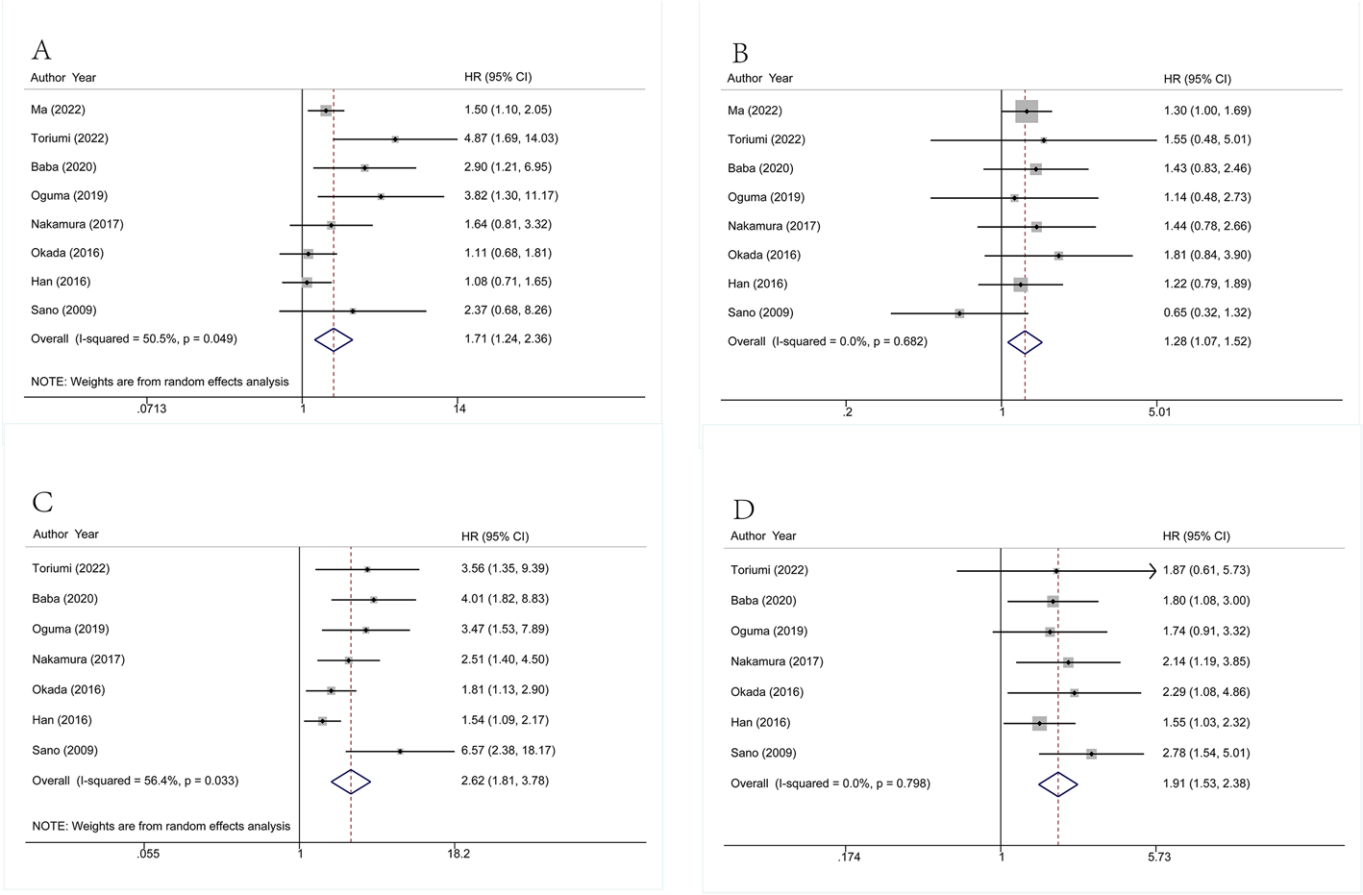
Forrest plot showing the pooled HR for RFS, (**A**) LI from multivariate analysis, (**B**) VI from multivariate analysis, (**C**) LI from univariate analysis, (**D**) VI from univariate analysis

Eight studies containing 1666 ESCC patients provided multivariate HR. The pooled multivariate HRs with 95% CIs for LI and VI were 1.71 (1.24–2.36, *p* = 0.001) and 1.28 (1.07–1.52, *p* = 0.07), respectively. The I^2^ for LI and VI was 50.5% (Chi^2^ = 14.15, *p* = 0.049) and 0% (Chi^2^ = 4.82, *p* = 0.682), respectively. Seven studies concerning 1270 patients provided univariate HR. The pooled univariate HRs with 95% CIs for LI and VI were 2.62 (1.81–3.78, *p* < 0.0001) and 1.91 (1.53–2.38, *p* < 0.0001), respectively. The I^2^ for LI and VI was 56.4% (Chi^2^ = 13.75, *p* = 0.033) and 0% (Chi^2^ = 3.09, *p* = 0.798), respectively. The pooled results are listed in Fig. 4.

### Publication bias

Begg’s test was used to test the publication bias of the included studies. In pooled multivariate OS, the *p value*s of publication bias for LI and VI were 0.017 and 0.596, respectively. In pooled univariate OS, the *p value*s of publication bias for LI and VI were 0.127 and 0.583, respectively. In pooled multivariate RFS, the *p value*s of publication bias for LI and VI were 0.063 and 1, respectively. In pooled univariate RFS, the *p value*s of publication bias for LI and VI were 0.035 and 0.368, respectively.

### Subgroup analysis of studies with haematoxylin–eosin (H&E) staining method

LI and VI were still poor prognostic parameter to OS in pooled multivariate and pooled univariate results. LI and VI were only poor indicator in RFS in pooled univariate analysis, not in pooled multivariate analysis. Detailed information was displayed in Supplementary Fig. 1, Supplementary Fig. 2, Supplementary Table 1 and Supplementary Table 2.

## Discussion

Our meta-analysis is the first study to explore the separate prognostic value of LI and VI in ESCC. A total of 27 articles with 5740 patients were enrolled. According to the pooled results of univariate analysis, distinguishing between the prognostic value of LI and VI for OS or RFS is difficult. VI and LI were both associated with adverse OS and RFS. The pooled multivariate results were more convincing than the pooled univariate results. Therefore, we focused on the pooled results from the multivariate analysis. The level of heterogeneity was acceptable for the pooled multivariate results. Both LI and VI were prognostic indicators of a poor OS. For RFS, the *p value* of VI was greater than 0.05. Therefore, we considered LI as a better predictor of a worse RFS than VI.

Many studies have focused on the relationship between LVI and prognosis in EC patients. The prognostic value of LVI in oesophageal cancer patients, N0 oesophageal cancer patients and superficial oesophageal cancer has been proven by our previous meta-analysis and many studies [4–6, 41–48]. Given the possible difference between LI and VI in prognosis, LI should be reported separately from VI according to the 8th AJCC Cancer Staging Manual [3]. An increasing number of studies have emphasized the prognostic value of LI and VI in EC patients. Zhang and his colleagues reported that the prognosis of patients with simultaneous LI and VI was worse than that of patients with LI or VI alone for thoracic ESCC. The 5-year overall survival of LI or VI alone, and simultaneous LI and VI was 54.5% and 33.9% respectively [10]. Tachezy et al. reported that LI was an independent prognostic indicator for both ESCC and adenocarcinoma patients, while VI was a significant factor for adenocarcinoma only [49]. The prognostic role of VI in EC patients is still controversial. Waraich et al. reported that VI was not a risk factor for oesophageal cancer recurrence [9]. Jia’s research suggested that VI was a favourable prognostic parameter in ESCC [50] Moreover, the prognostic value of LI and VI in N0 oesophageal carcinoma patients was not consistent among studies [7, 9, 49, 51, 52]. Due to the uncertain prognostic value of VI and LI in EC patients, a meta-analysis was essential to clarify the problem.

In the current meta-analysis, LI was an indicator of a poor OS and RFS; however, VI was a poor indicator only for OS. How to interpret such results? It could well be due to a type-2 error that vascular invasion did not show a statistical significance with RFS in the current study. Additionally, LI has a more significant role in prognosis in the early stages, such as stage I and stage II oesophageal cancer, than VI. However, the prognostic role of VI is more significant than LI in stage III oesophageal cancer [43]. The pathological information of LI and VI was acquired from surgical specimen. The majority of patients in the enrolled studies were in early stage (stage I and stage II). This may be the reason why LI was more significant than VI in our meta-analysis.

The distinction of blood vessels from lymphatic vessels is made by the presence of erythrocytes in the endothelial line and thick vessel walls. However, the distinction between LI and VI is not obvious by haematoxylin–eosin (H&E) staining [7]. The positive rate of LI, VI or LVI staining by immunohistochemistry (IHC) is higher than that of the H&E staining method in the same group of patients [7, 53, 54]. The IHC method of staining the vascular endothelium (CD34) and the lymphatic endothelium (podoplanin) could increase the possibility of distinguishing lymphatic and vascular invasion [53, 55]. Due to limited number of studies with IHC staining method, we conducted a subgroup analysis of studies with H&E staining method. Pooled results of RFS indicated that the prognostic role of LI and VI was weakened. LI and VI were only poor indicator in RFS in pooled univariate analysis, not in pooled multivariate analysis. This may be explained by the fact that HE staining method is not as specific or sensitive as IHC staining method. However, only three of included papers used IHC staining method and many of the included studies did not provide the staining method. We advocate that IHC staining method should be used when LI and VI need to be separated in future studies.

There were also some limitations of this meta-analysis. First, the studies included were restricted to papers published in English. This may lead to some potential bias. Actually, the *p value* was less than 0.05 for the publication bias of multivariate LI. Second, tumour stage and staining methods that could lead to different positive rates of LI and VI were not evaluated by the same method among the included studies. Studies investigating the prognostic value of LI and VI should apply the IHC method. Third, I^2^ was all < 50% which indicated moderate heterogeneity except the I^2^ of RFS in pooled results of univariate analysis. The heterogeneity among studies should be noticed although they were acceptable. The heterogeneity could influence the credibility of the results. It must be resolved appropriately when the heterogeneity is medium and extreme. Last but not least, the present study only focused on ESCC, therefore the results should be interpreted with caution in western countries.

## Conclusions

We hold the opinion that LI and VI are indicators of poor OS in ESCC patients. LI predicts a worse RFS in ESCC patients. Compared to VI, LI is a more significant indicator of a worse RFS. More large-sample studies with immunohistochemical staining and good designs are required to detect the prognostic value of separate LI and VI in ESCC.

## Supplementary Information

**Additional file 1: Supplementary Figure 1.** Forrest plot showing the pooled HR for OS, (A) LI from multivariate analysis, (B) VI from multivariate analysis, (C) LI from univariate analysis, (D) VI from univariate analysis.

**Additional file 2: Supplementary Figure 2.** Forrest plot showing the pooled HR for RFS, (A) LI from multivariate analysis, (B) VI from multivariate analysis, (C) LI from univariate analysis, (D) VI from univariate analysis.

**Additional file 3: Supplementary Table 1.** Pooled results of multivariate analysis. **Supplementary Table 2.** Pooled results of univariate analysis.

**Additional file 4.**

## Data Availability

All data generated or analysed during this study are included in this published article and its supplementary information files.
