# Supplementary material for: The prognostic value of separate lymphatic invasion and vascular invasion in oesophageal squamous cell carcinoma: a meta-analysis and systematic review
Source: BMC Cancer. 2022 Dec 19;22:1329. doi: 10.1186/s12885-022-10441-6 (PMC9764535; doi:10.1186/s12885-022-10441-6)
Supplement: Supplementary file 1 — Additional file 1: Supplementary Figure 1. Forrest plot showing the pooled HR for OS, (A) LI from multivariate analysis, (B) VI from multivariate analysis, (C) LI from univariate analysis, (D) VI from univariate analysis. [file 12885_2022_10441_MOESM1_ESM.docx]

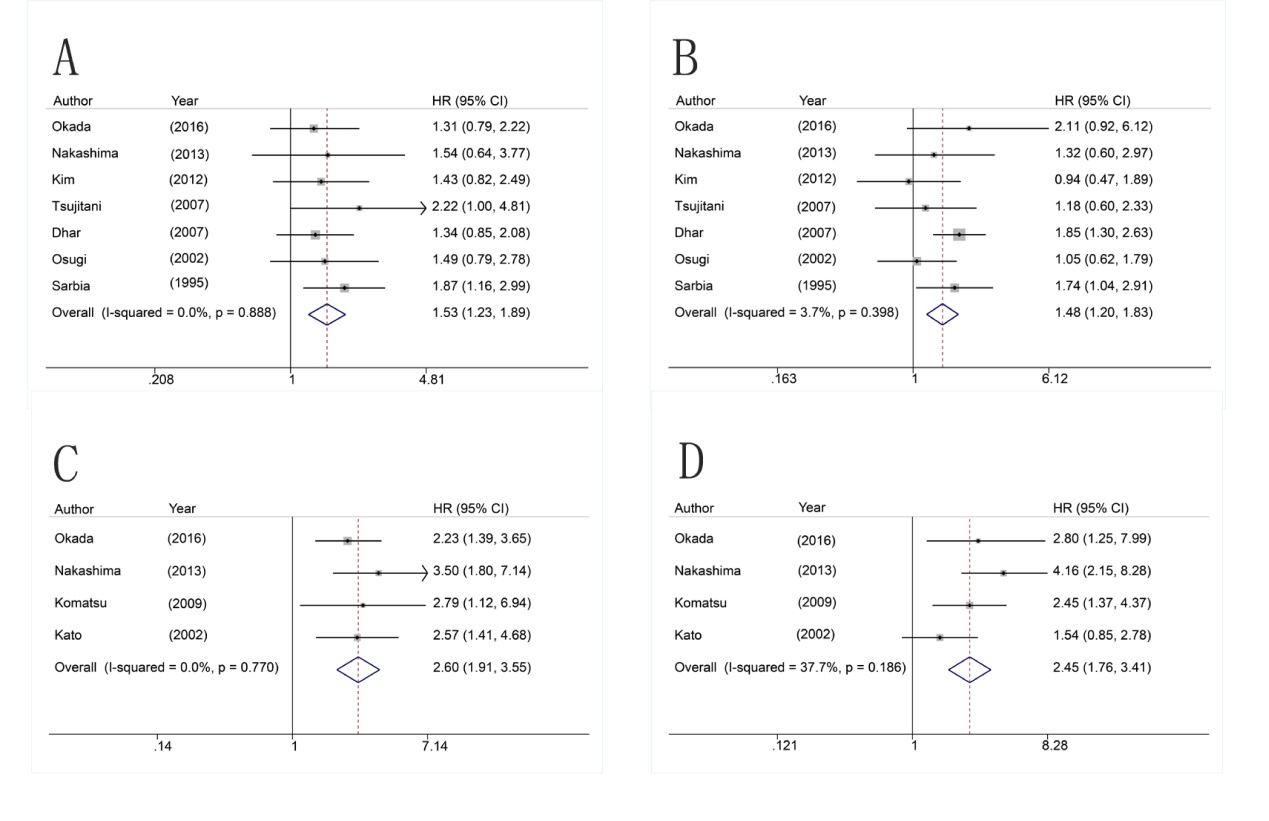


Supplementary Figure 1: Forrest plot showing the pooled HR for OS, (A) LI from multivariate analysis, (B) VI from multivariate analysis, (C) LI from univariate analysis, (D) VI from univariate analysis
