# Supplementary material for: The prognostic value of separate lymphatic invasion and vascular invasion in oesophageal squamous cell carcinoma: a meta-analysis and systematic review
Source: BMC Cancer. 2022 Dec 19;22:1329. doi: 10.1186/s12885-022-10441-6 (PMC9764535; doi:10.1186/s12885-022-10441-6)
Supplement: Supplementary file 3 — Additional file 3: Supplementary Table 1. Pooled results of multivariate analysis. Supplementary Table 2. Pooled results of univariate analysis. [file 12885_2022_10441_MOESM3_ESM.docx]

Supplementary Table 1 Pooled results of multivariate analysis

| Indicator | LI | | Heterogeneity analysis | | Publication bias | VI | | Heterogeneity analysis | | Publication bias |
| --- | --- | --- | --- | --- | --- | --- | --- | --- | --- | --- |
|  | HR (95% CI) | *p* value | I^2^ | *p* value |  | HR (95% CI) | *p* value | I^2^ | *p* value |  |
| OS | 1.53 (1.23-1.89) | <0.0001 | 0% | 0.888 | 0.23 | 1.48 (1.2-1.83) | <0.0001 | 3.7% | 0.398 | 1 |
| RFS | 1.9 (0.84-4.31) | 0.123 | 58.5% | 0.09 | 1 | 1.03 (0.65-1.63) | 0.912 | 39.9% | 0.19 | 1 |

Supplementary Table 2 Pooled results of univariate analysis

| Indicator | LI | | Heterogeneity analysis | | Publication bias | VI | | Heterogeneity analysis | | Publication bias |
| --- | --- | --- | --- | --- | --- | --- | --- | --- | --- | --- |
|  | HR (95% CI) | *p* value | I^2^ | *p* value |  | HR (95% CI) | *p* value | I^2^ | *p* value |  |
| OS | 2.6 (1.91-3.55) | <0.0001 | 0% | 0.77 | 0.308 | 2.45 (1.76-3.41) | <0.0001 | 37.7% | 0.186 | 0.734 |
| RFS | 3.11 (1.48-6.55) | 0.0003 | 65.8% | 0.054 | 0.296 | 2.25 (1.53-3.32) | <0.0001 | 0% | 0.577 | 1 |
